# Triptolide Combined with Radiotherapy for the Treatment of Nasopharyngeal Carcinoma via NF-κB-Related Mechanism

**DOI:** 10.3390/ijms17122139

**Published:** 2016-12-19

**Authors:** Weiying Zhang, Min Kang, Tingting Zhang, Bo Li, Xueyin Liao, Rensheng Wang

**Affiliations:** Department of Radiation Oncology, The First Affiliated Hospital of Guangxi Medical University, 6 Shuangyong Road, Nanning 530021, China; zwy646@163.com (W.Z.); km1019@163.com (M.K.); ztt870920@sina.com (T.Z.); xintouboshi@gmail.com (B.L.); liaoxueyin91@163.com (X.L.)

**Keywords:** triptolide, ionizing radiation, nasopharyngeal carcinoma, NF-κB, phosphorylation

## Abstract

Advanced nasopharyngeal carcinoma (NPC) has a poor prognosis because of the lack of an effective treatment. Here we explored the efficiency and the molecular mechanisms of combined treatment with triptolide and ionizing radiation for treating NPC. Human nasopharyngeal carcinoma (CNE) cells were treated with triptolide, ionizing radiation, or triptolide plus ionizing radiation in vitro. Tumor potency was examined in an in vivo CNE cell xenograft mouse model, which was treated as above. Our results demonstrated that triptolide caused a significant reduction in cell growth and colony number, and induced a marked apoptosis that was further enhanced with increasing doses of ionizing radiation. Combination treatment synergistically reduced tumor weight and volume without obvious toxicity. Western blot analysis in vitro and in vivo showed that triptolide induced apoptotic protein Bax expression and inhibited phosph-NF-κB p65, Bcl-2 and VEGF proteins without affecting other NF-κB related protein expression. In conclusion, our findings revealed that triptolide plus ionizing radiation had synergistic anti-tumor and anti-angiogenesis effects in NPC via down-regulating NF-κB p65 phosphorylation. The combination therapy may provide novel mechanism insights into inhibit NPC.

## 1. Introduction

Nasopharyngeal carcinoma (NPC) is a rare malignant disease that is found worldwide but is highly prevalent in South China, reaching a peak incidence of around 20–30 per 100,000 cases [1,2]. The local invasiveness and metastasis is a common clinical problem for NPC. At present, the standard treatment is a combination treatment with radiotherapy and chemotherapy. However, owing to the frequency of local recurrence, the distant metastasis, long-term secondary effects of radiotherapy and chemotherapy, the five-year overall survival rate is only 50%–60% [3]. In addition, due to toxicity, the side effects, and even increased incidence of late complications without obvious survival benefits, these methods are hard to complete [4]. It is, therefore, clinically desirable to develop treatments to decrease its invasive and metastatic potential. Currently, some studies indicated that natural, synthetic, or biological chemicals had effective cancer chemoprevention in the prevention, suppression, or delay of the carcinogenesis process with fewer side effects [5,6].

Triptolide (TPL) is a diterpenoid triepoxide derived from the Chinese herb *Tripterygium wilfordii* (Celastraceae). It has been reported to have various pharmacological effects, such as anti-inflammation, anti-oxidant, and anti-angiogenesis properties. In particular, the anti-cancer activity of TPL has attracted the most interest [7,8]. In previous studies, the anti-tumor effect has been demonstrated in many malignant diseases [9,10,11,12]. Furthermore, it was found that TPL, combined with other anti-cancer agents and ionizing radiation (IR), synergistically increased their cytotoxic effects, suggesting it to be a combinatorial drug for the treatment of malignancies [13,14,15,16].

The nuclear factor-κB (NF-κB) family includes NF-κB1 (p50), NF-κB2 (p52), c-Rel, RelA/p65, and RelB. NF-κB p65 (p65) is a key component of NF-κB activation and plays an important role in regulating multiple biological functions, including inflammation, immunity, cell proliferation, apoptosis, and tumor migration [17]. p65 resides in the cytoplasm in an inactive form by bounding to its inhibitory proteins called IB-κ in the normal cells. However, high level of p65 activity is found in many human cancer cells including hepatocellular carcinoma, sporadic colorectal cancer, ovarian cancer, gastric carcinoma, and so on [18,19,20,21,22,23]. Now, p65 has been considered as a well-established marker of tumor progression, as well as a poor prognosis factor for survival, and represents a useful target for the management of these cancers. Thus, suppression of p65 should be effective to induce apoptosis of tumor cells. Many previous studies prove that NF-κB, especially the p65 subunit, plays an important role in NPC development [24,25,26,27]. In addition, angiogenesis and invasion are characteristic features of malignant neoplasms and play prominent roles in the progression of cancer [28,29]. Many molecular events may be involved in the angiogenesis of malignant tumors, including NPC, but recent intensive studies have focused on the key role of vascular endothelial growth factor (VEGF) [30]. VEGF has been proved to be not only a promoter of invasion via directly disrupting endothelial barrier function, but also a major mediator of angiogenesis via regulating majority steps in the angiogenic cascades [31,32,33]. It is noteworthy that the endogenous VEGF is controlled by multiple important machineries, among which the NF-κB pathway is a pivotal one [34]. Therefore, it seems reasonable to hypothesize that targeting the NF-κB pathway may provide a novel therapeutic modality for inhibiting cancers with significant angiogenesis and growth.

It has been shown that TPL exerts its anti-cancer properties by regulating the NF-κB signal pathways [35,36]. However, the effects of TPL combined with radiotherapy on NPC remain unknown. The present study is designed to determine the combined efficacy of TPL and radiotherapy on NPC in vitro and in vivo, and to investigate the synergistic cytotoxicity on the apoptosis-inducing and anti-angiogenesis effects. Furthermore, to uncover the molecular mechanisms, the involvement of the NF-κB signal pathways in mediating TPL/IR-triggered anti-tumor effect were investigated to help us to better use TPL in cancer therapy.

## 2. Results

### 2.1. Effects of Combined Therapy on CNE Cell Proliferation

**Figure 1:**
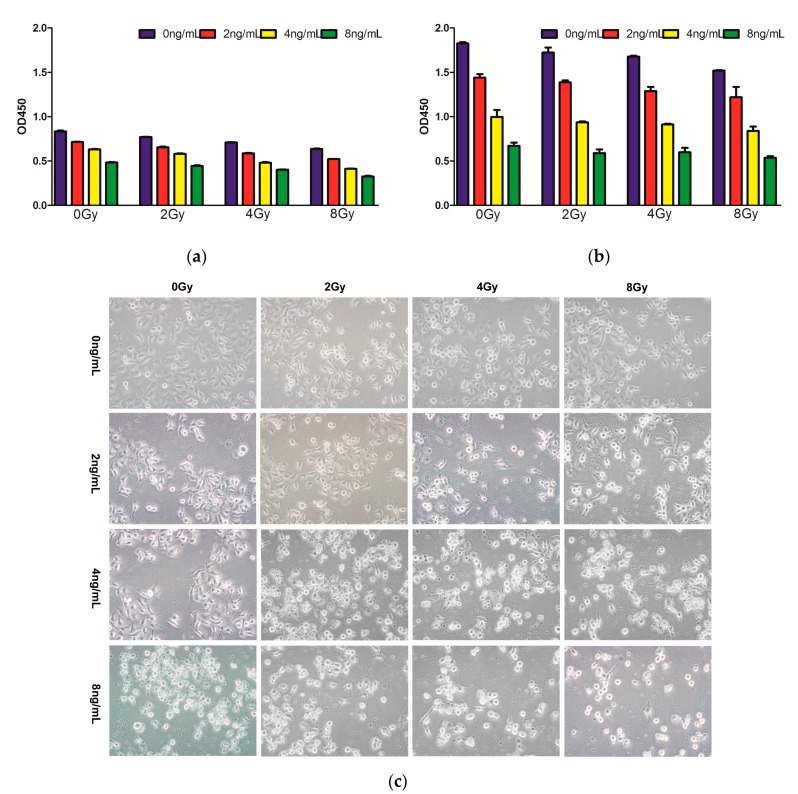
CCK-8 assay of combined treatment with Triptolide (TPL) (0, 2, 4, or 8 ng/mL) and IR (0, 2, 4, or 8 Gy) on CNE cell proliferation in vitro on day 1 (**a**) and day 4 (**b**); Morphological observation (**c**) of CNE cells by treatment with TPL (0, 2, 4, or 8 ng/mL), IR (0, 2, 4, or 8 Gy), or TPL plus IR.

To investigate the effects of TPL and IR treatment on CNE cell proliferation, the CCK-8 assay was performed. CNE cells were treated with TPL, IR, TPL, and IR, respectively, for one and four days, followed by cell growth measurement. Our results indicated that, at both day 1 (Figure 1a) and day 4 (Figure 1b), TPL and IR both inhibited the proliferation of CNE cells and the cell proliferation was decreasing as the treatment concentration of TPL and doses of IR increased. TPL alone inhibited cell proliferation more significantly than IR alone. Morphological observation (Figure 1c) indicated that after 48 h of incubation, compared with control group, CNE cells treated with TPL and IR became round, smaller, bloated, and deformed. The changes became more serious as the treatment concentration of TPL and doses of IR increased, and TPL alone changed cell morphology more significantly than IR alone. The combined treatment had a synergistic inhibitory effect.

### 2.2. Effects of Combined Therapy on CNE Cell Clonogenicity

**Figure 2:**
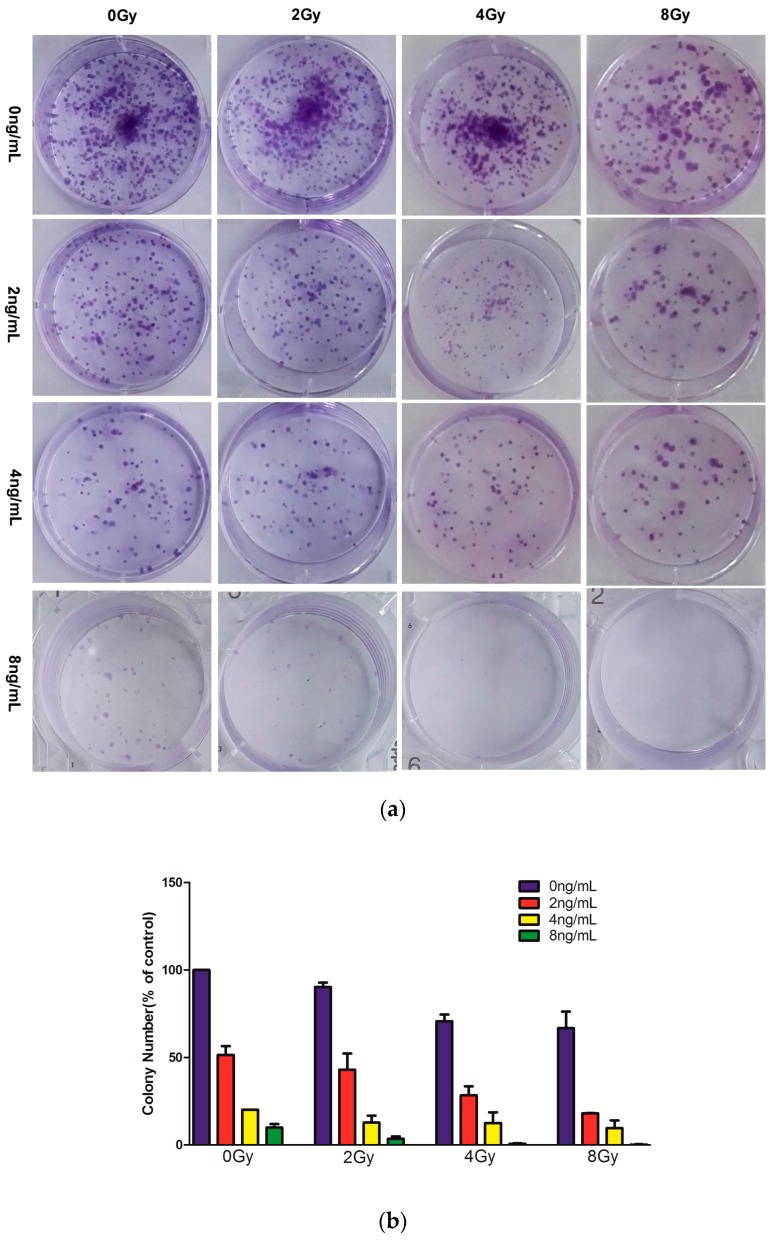
CNE cell clonogenicity (**a**) by treatment with TPL (0, 2, 4, or 8 ng/mL), IR (0, 2, 4, or 8 Gy), or TPL plus IR in vitro; and (**b**) histogram representing the surviving fraction of CNE cells.

We first investigated whether combined treatment with TPL and IR inhibited clonogenicity in vitro. Figure 2 showed that IR alone (0, 2, 4, or 8 Gy) or in combination with 0, 2, 4, or 8 ng/mL TPL inhibited clonogenic formation in CNE cells. Seven days after IR, the surviving fraction were 100%, 90.2%, 70.7%, and 66.7% for 0, 2, 4, and 8 Gy, respectively. The surviving fraction were 51.5%, 43%, 28.4%, and 18.1% after TPL (2 ng/mL) plus IR at 0, 2, 4, and 8 Gy, respectively. The surviving fraction were 20.1%, 12.8%, 12.6%, and 9.7% after TPL (4 ng/mL) plus IR at 0, 2, 4, and 8 Gy, respectively. When TPL concentration reached 8 ng/mL, almost no colonies were formed. These results indicated that both TPL and IR inhibited colony formation and that the combined treatment had a synergistic inhibitory effect.

### 2.3. Effects of TPL Combination Treatments on Apoptotic Process in CNE Cells

**Figure 3:**
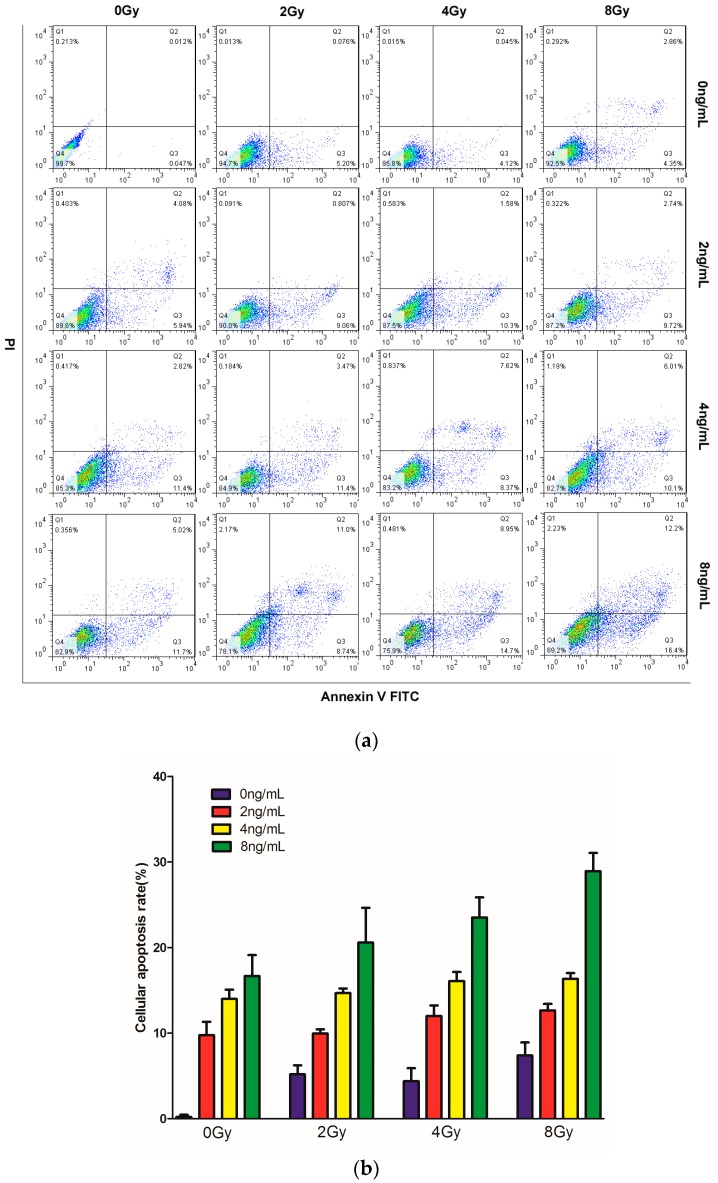
Apoptotic process (**a**) of CNE cells by treatment with TPL (0, 2, 4, or 8 ng/mL), IR (0, 2, 4, or 8 Gy), or TPL plus IR in vitro. Annexin V/PI staining was performed; and (**b**) histogram representing the percentage of annexin V-positive cells.

To test whether combined TPL and IR treatment enhanced cell apoptosis in vitro, CNE cells were treated with TPL, IR, or both, and annexin V-FITC/PI double staining was performed (Figure 3a). In the cells treated with 2, 4, and 8 Gy IR alone, the apoptosis rate was 5.27%, 4.16%, and 7.21%, respectively, whereas in the cells treated with 2, 4, and 8 ng/mL TPL alone, the apoptosis rate was 10%, 14.32%, and 16.72%, respectively (Figure 3b). TPL can induce more cell apoptosis than IR alone. Moreover, TPL enhanced IR treatment on cell apoptosis. After TPL (8 ng/mL) plus IR at 2, 4, and 8 Gy, the apoptosis rate was 19.74%, 23.65%, and 28.6%, respectively. The results showed that the apoptosis rate of the combined treatment was significantly higher than the corresponding IR or TPL treatment alone. These results indicated that TPL plus IR had a synergistic effect and enhanced apoptosis induction in vitro.

### 2.4. Effects of TPL Combination Therapy on Tube Formation of HUVECs

**Figure 4:**
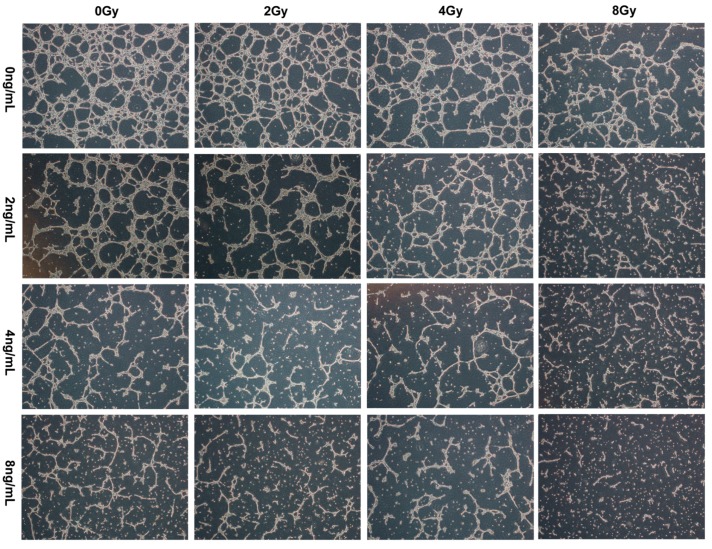
The tube formation of HUVECs by treatment with TPL (0, 2, 4 or 8 ng/mL), IR (0, 2, 4 or 8 Gy), or TPL plus IR in vitro.

To test whether combined TPL and IR treatment had anti-angiogenic activities in vitro, the tube formation of HUVECs were detected. The results in Figure 4 indicated that in the vehicle group, HUVECs developed capillary/tube-like structures with interconnecting networks. Compared with the vehicle group, TPL and IR both reduced the extent of tubular formation of HUVECs and the network formation was decreasing as the treatment concentration of TPL and doses of IR increased. TPL alone inhibited angiogenesis more significantly than IR alone. The combined treatment had a synergistic anti-angiogenic effect.

### 2.5. Effects of TPL Combination Therapy on Related Protein Expression Levels in CNE Cells

**Figure 5:**
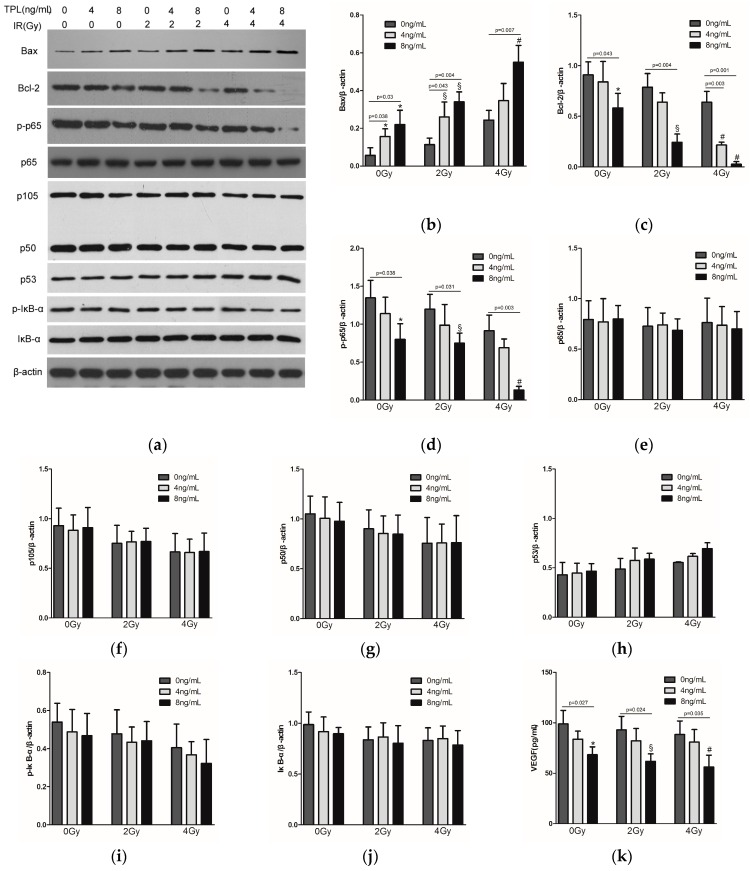
(**a**) Western blot assay for Bax, Bcl-2, phosph-p65, and p65 expression in vitro with combined treatment with TPL and IR. Densitometry of Bax (**b**), Bcl-2 (**c**), phosph-p65 (**d**), p65 (**e**), p105 (**f**), p50 (**g**), p53 (**h**), phosph-IκB-α (**i**), and IκB-α (**j**) from Western blot experiment; (**k**) ELISA analysis for VEGF secretion in vitro with combined treatment with TPL and IR. (*n* = 3, * *p* < 0.05 compared with control group; § *p* < 0.05 compared with 2 Gy IR treatment group; and # *p* < 0.05 compared with 4 Gy IR treatment group).

To explore the cellular mechanisms for the suppression of CNE cells by TPL and IR, the expression of p65, phosph-p65, Bcl-2, and Bax in CNE cells were examined by Western blot, with β-actin as protein loading control (Figure 5a). As shown in Figure 5b, the expression of Bax in the groups treated with 8 ng/mL TPL plus 0, 2, or 4 Gy IR, respectively, was significantly higher than in the groups treated with 0, 2, or 4 Gy IR alone (*p* < 0.05). On the contrary, Bcl-2 proteins in the groups treated with 8 ng/mL TPL plus 0, 2, or 4 Gy IR, respectively, were significantly fewer than in the groups treated with 0, 2, or 4 Gy IR alone (*p* < 0.05) (Figure 5c). Similarly, in Figure 5d, TPL significantly decreased the expression of phosph-p65. When combined with IR (4 Gy), the expression of phosph-p65 proteins was lowest compared to the other groups (*p* < 0.05). However, Figure 5e showed that there was no significant difference in p65 expression between untreated control group and the experimental groups, meaning that neither IR nor TPL affected p65 expression. In addition, other NF-κB related proteins, such as p105, p50, p53, p-IκB-α, and IκB-α (Figure 5f,g), were tested, the results indicated that there was no significant difference in the expression of these proteins between untreated control group and the experimental groups, also meaning that neither IR nor TPL affected these proteins. ELISA analysis also revealed 8 ng/mL TPL plus 0, 2 or 4 Gy IR significantly decreased protein levels of VEGF (Figure 5k), but 4 ng/mL TPL and IR did not. By comparing all target proteins, TPL plus IR significantly reduced phosph-p65, VEGF, and Bcl-2 proteins levels in vitro.

### 2.6. In Vivo Animal Study

**Figure 6:**
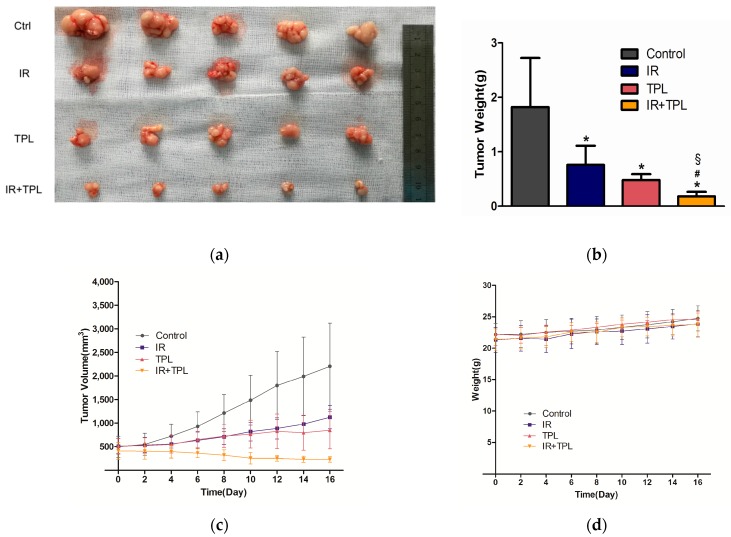
Combined treatment with TPL and IR suppressed tumor growth in a human NPC nude mouse model. (**a**) Gross evaluation of tumor volumes in the control group, IR treatment group, TPL treatment group, and combination treatment group on day 16; (**b**) the weight of the tumors from the mice in four groups; (**c**) tumor volumes measured in situ every other day in four groups; and (**d**) weight changes of the nude mice were measured after treatment every other day; (*n* = 5; * *p* < 0.05 compared with the control group; § *p* < 0.05 compared with the IR treatment group; and # *p* < 0.05 compared with the TPL treatment group).

To directly evaluate the synergistic inhibitory effect of TPL and IR on tumors in vivo, tumor growth of CNE cells in nude mice were tested using a tumor xenograft model. After the mice were sacrificed, the tumors were removed from the mice and pictures were taken. The gross evaluation (Figure 6a) showed that greater in vivo tumor formation was observed in the control mice compared to mice treated with TPL, IR, or in combination, and the smallest tumors were observed in the combination group. Figure 6b showed that the final weight of the tumors was significantly heavier in the control group than in the experimental groups, and the lightest tumors were observed in the combination group (*p* < 0.05). During the experiment, greater tumor volume was observed in the control group compared to the experimental groups, and the tumor growth was slowest in the combination group (Figure 6c). No significant weight changes in the mice were observed during the treatment (Figure 6d). These results suggested that none of the treatments induced significant weight loss and had obvious toxicity. These results revealed that TPL had a strong synergistic inhibitory effect on tumor growth when used in combination with IR on CNE cells in vivo.

**Figure 7:**
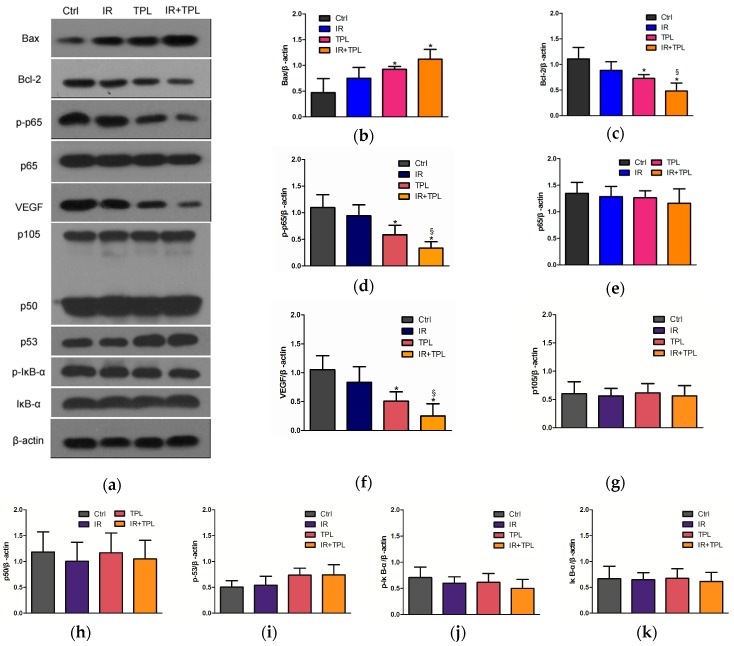
(**a**) Western blot assay for Bax, Bcl-2, phosph-p65, p65, and VEGF expression in vivo with combined treatment with TPL and IR. Densitometry of Bax (**b**), Bcl-2 (**c**), phosph-p65 (**d**), p65 (**e**), VEGF (**f**), p105 (**g**), p50 (**h**), p53 (**i**), phosph-IκB-α (**j**), and IκB-α (**k**) from Western blot experiments. (*n* = 5; * *p* < 0.05 compared with the control group; and § *p* < 0.05 compared with the IR treatment group).

To determine whether apoptosis and anti-angiogenesis was involved in the TPL-enhanced regression of CNE tumors, meanwhile, to explore whether TPL affected NPC via inhibiting p65 activity in vivo, Western blot assays were performed. The expression of p65, phosph-p65, Bcl-2, Bax, and VEGF in tumors after 16 days was examined, with β-actin as the loading control (Figure 7a). As shown in Figure 7a,b, Bax expression was significantly higher in the combined treatment group and TPL treatment group than in the untreated control group (*p* < 0.05). On the contrary, Bcl-2 expression was the lowest in the combined treatment group (Figure 7a,c) (*p* < 0.05). As shown in Figure 7a,e,g–k, there were no significant differences in p65, p105, p50, p53, p-IκB-α, and IκB-α expression in all groups. However, phosph-p65 expression of the group receiving combined treatment was significantly lower than that of the control group and IR treatment group (Figure 7a,d) (*p* < 0.05). In addition, phosph-p65 expression in TPL treatment group was also significantly lower than that in the control group (Figure 7a,d) (*p* < 0.05). Meanwhile, Western blot analysis also revealed significantly decreased protein levels of VEGF (Figure 7a,f) in the combined treatment group compared with the control group and IR treatment group (*p* < 0.05). These data indicated that TPL induced apoptotic protein expression and decreased anti-apoptotic and angiogenesis protein expression, as well as TPL plus IR enhanced apoptotic and anti-apoptotic effects in vivo. Interestingly, the change trends of Bcl-2 and VEGF expression in the four groups were largely consistent with those of phosph-p65 expression. This seemed to indicate that TPL inhibited Bcl-2 and VEGF expression by mediating down-regulation of phosph-p65 expression.

## 3. Discussion

Radiation therapy, alone or in combination with surgical resection or current chemotherapy, has demonstrated efficacy in local tumor and non-resectable tumors in patients with advanced NPC. However, these methods caused side effects, or even increased the incidence of late complications.

Triptolide is such a small, soluble, stable, and multifunctional molecule that could be easily produced and conveniently delivered. In addition, use of this small molecule bypasses the vast majority of inconvenience and disadvantage associated with protein and gene therapy, such as poor permeability, immunogenicity, low efficacy, high cost, and so on. Third, TPL has been successfully used in the clinical treatment of nephritic syndrome, rheumatoid arthritis, idiopathic pulmonary fibrosis, organ transplant, and so on, without obvious side effects [37]. In addition, it has been shown to act synergistically with the conventional chemotherapeutic drugs in some cancers [12,13,38]. It is now known that TPL has an anti-cancer effect on NPC. [39] Another previous study showed that TPL, in combination with IR, produced synergistic anti-tumor effects in oral cancer [40]. Therefore, combining this potential herb-derived drug with other adjuvant therapy for patients with advanced NPC is worth considering.

To effectively use TPL for clinical development, it is essential to understand its mode of actions and potential biomarkers. Some previous studies have shown that TPL can inhibit cancer cell proliferation and induce cancer cell apoptosis [8]. In our study, TPL and IR inhibited the proliferation of CNE cells and the cell proliferation was decreasing as the treatment concentration of TPL and doses of IR increased. The percentage of cells positive for only annexin V increased to 28.6% following treatment with 8 ng/mL TPL in combination with 8 Gy IR (Figure 3a). These results indicated that TPL plus IR increased apoptosis induction in vitro.

The Bcl-2 family of proteins, located on the mitochondrial membrane, promote (Bax, Bak, Bad) or inhibit apoptosis (Bcl-2, Bcl-XL) through regulating the release of cytochrome C from the mitochondria into the cytoplasm [41]. In our study, the results in vitro showed that the expression of Bcl-2 decreased and Bax increased after TPL treatment. Our results of the apoptosis effect of TPL were similar with previous study [11] about TPL on human anaplastic thyroid carcinoma cells. We speculated that TPL plus IR enhanced the induction of apoptosis by increasing the levels of apoptotic proteins in vitro. TPL had a fairly strong impact, while radiation showed a very slight effect (Figure 5a). Combination treatments have significant differences on cell apoptosis compared with control and single treatments, moreover, the synergistic effect had a concentration-dependent manner, that is to say, the higher the concentration of TPL and the dose of IR were, the stronger the synergistic effect was. In addition, in vivo study, when compared with the mice undergoing control treatment or single treatment, the mice undergoing combined treatment showed a marked and significant decrease in tumor weight (Figure 6b). Western blot revealed that TPL induced apoptotic protein (Bax) and decreased anti-apoptotic protein (Bcl-2) expression in xenograft tumor tissues. Furthermore, combination treatment had a better anti-apoptotic effect than single treatment (Figure 7a).

The central role of VEGF in pathologic angiogenesis and invasion, combined with its restricted expression in healthy adults, has spurred the development of a variety of therapeutic strategies aimed at blocking VEGF for antitumor therapy [42]. In some cancers, TPL has been indicated to inhibit VEGF [35,43]. In this study, we explored the anti-angiogenesis effect of TPL on NPC via blocking VEGF. The results, in vitro, showed that 8 ng/mL TPL plus IR decreased VEGF protein expression significantly, but 4 ng/mL TPL or/and IR had no significant effect on VEGF expression. In addition, the results in vivo showed that TPL alone and TPL plus IR decreased VEGF protein expression significantly, meaning that TPL inhibited angiogenesis of NPC and IR plus TPL enhanced this effect.

Accumulating evidence suggested that the inhibition of NF-κB could repress tumor angiogenesis and tumorigenesis through down-regulating its downstream gene expression [44,45]. It was known that activation of the signaling pathway can result in modulation of target proteins, as well as activation of transcription factors, leading to cellular alterations. The activation of NF-κB was usually thought to require degradation of IκB. However, it was found that post-translational modifications of the subunits of NF-κB determined the functional activity. Serine 536 of p65 is a target of multiple protein kinases, like IκB kinase α/β (IKKα/β). Phosphorylation of p65 has been reported to suppress nuclear export of NF-κB and increase transactivation of many downstream genes through positive interaction of p65 with co-activators [46,47,48,49,50]. It also activated the survival-promoting pathway when cells were challenged with chemotherapeutic drugs [51,52]. When p65 was activated, in other words, phosphorylated p65 could translocate to the nucleus and associate with the promoter regions of multiple target genes [53]. In this study, we discovered that TPL was able to decrease p65 phosphorylation without affecting p65 expression, as well as further potentiating the inhibition of NF-κB activity. However, the expression of p105, p50, p53, p-IκB-α, and IκB-α had no significant difference in all groups, like p65 expression. That is to say, TPL only affected p65 phosphorylation in NF-κB pathway. Meanwhile, VEGF and Bcl-2 expression were significantly decreased in TPL treated group. In addition, IR alone cannot significantly affect all target protein expression compared with the untreated group. Thus, VEGF and Bcl-2 expression were significantly related to p65 phosphorylation. We speculated that TPL induced anti-angiogenesis activities in CNE cells through a mechanism that inhibits p65 activation, leading to down-regulation of a number of NF-κB targeting genes, including VEGF, Bcl-2, and Bax.

## 4. Materials and Methods

### 4.1. Cell Culture and Drug Treatment

Human nasopharyngeal carcinoma (CNE) cells (purchased from Shanghai Institute of Cell Bank, Chinese Academy of Science), were cultured in RPMI medium (Gibco, Grand Island, NY, USA) containing 10% fetal bovine serum (FBS, Gibco, Carlsbad, CA, USA), penicillin (100 U/mL), and streptomycin (100 U/mL), in culture dishes at 37 °C in a humidified atmosphere with 5% CO_2_. TPL (purity is more than 98%, Medchem Express, NJ, USA) was dissolved in dimethylsulfoxide (DMSO) as a 4 mg/mL stock and added to cells at the indicated concentrations.

### 4.2. Cell Proliferation Assay

Briefly, CNE cells at the logarithmic growth phase were seeded onto 96-well culture plates at a density of 2 × 10^3^ cells/well. After being incubated for 24 h, these cells were subjected to vehicle (DMSO), the TPL treatment at different concentrations (0, 2, 4, or 8 ng/mL, respectively); IR at a dose of 0, 2, 4, or 8 Gy; or TPL plus IR, and incubated for one and four days. At different time points, Cell Counting Kit-8 (CCK-8, Dojindo, Tokyo, Japan) was mixed with culture medium at 10% of the volume and incubated with cells for 4 h. Absorbance at 450 nm for each well was measured using a spectrophotometer (Synergy 2, BioTek, Winooski, VT, USA).

### 4.3. Morphological Observation

CNE cells in logarithmic growth were seeded at 1 × 10^5^/well in a 12-well plate, After adhesion for 24 h, and cells were treated with vehicle (DMSO) alone; TPL (0, 2, 4, or 8 ng/mL); IR (at a dose of 0, 2, 4, or 8 Gy); or TPL plus IR for 48 h before observation with an inverted microscope (Olympus, Tokyo, Japan).

### 4.4. Colony Formation Assay

CNE cells were seeded into a six-well culture dish (1000 cells/well). After incubation for 24 h, cells were treated with vehicle (DMSO) alone; TPL (0, 2, 4, or 8 ng/mL); IR (at a dose of 0, 2, 4, or 8 Gy); or TPL plus IR. After seven days, the cells were washed twice with PBS, and fixed for 30 min at room temperature with 4% paraformaldehyde. The colonies (containing z50 cells) were stained with crystal violet, and the number of colonies was counted using a microscope.

### 4.5. Cell Apoptosis Assay

CNE cells were seeded into six-well plates at a density of 1 × 10^6^ cells/well. Exactly 48 h after treatment with TPL (0, 2, 4, or 8 ng/mL), IR (0, 2, 4, or 8 Gy), or TPL plus IR, cells were harvested. First, the cells were washed in binding buffer and centrifuge at 300× *g* for 10 min. The supernatant was aspirated completely; Second, 10 µL of Annexin V-FITC per 10^6^ cells was added and mixed with cells well and incubated for 15 min in the dark at room temperature; Third, the cells were washed and the supernatant was aspirated; Finally, 5 µL of PI solution was add immediately prior to analysis by flow cytometry or fluorescence microscopy, samples were then measured by flow cytometry (FACS; Becton Dickinson, Coppell, TX, USA). The data were analyzed with CellQuest software.

### 4.6. Tube Formation Assay

The 96-well plate was coated with Matrigel (10 mg/mL) and incubated at 37 °C with 5% CO_2_ for 30 min. Then, HUVEC cells were seeded into 96-well plate at a density of 1 × 10^4^ cells/well. Exactly 48 h after treatment with TPL (0, 2, 4, or 8 ng/mL), IR (0, 2, 4, or 8 Gy), or TPL plus IR, the capillary-like tube formation of HUVEC cells in each well was photographed with an inverted microscope.

### 4.7. Western Blot Analysis and ELISA Analysis

CNE cells were seeded into six-well plates at a density of 1 × 10^6^ cells/well. For exploring the mechanism of TPL plus IR, especially TPL on inhibiting CNE cells, based on the above assays, the treatment of TPL (0, 4, or 8 ng/mL), IR (0, 2, or 4 Gy), or TPL plus IR was used on CNE cells. Exactly 48 h after the treatment, cells were harvested. The cells were collected and homogenized in 200 mL of ice-cold RIPA (Bio-Rad, Hercules, CA, USA) supplemented with 1 μL of 200 mM PMSF (Kang Chen Corp., Shanghai, China), respectively. The mixture was incubated on ice for 60 min and then centrifuged at 12,000 rpm for 10 min. The supernatant was collected and the protein concentrations of the cell lysates were measured by the BCA protein assay kit (Thermo, Rockford, IL, USA). The samples were electrophoresed through a 10% SDS-PAGE gel, and then transferred onto PVDF membranes (Millipore, Bedford, MA, USA). The membranes were then blocked in 5% non-fat milk in TBST buffer (50 mm Tris-HCl, 100 mm NaCl, and 0.1% Tween-20, pH 7.4) for one hour at room temperature. After blocking, the membranes were washed three times with TBST. Then, the membranes were incubated with antibodies against p65 (Cat.: #8242, Cell Signaling, Beverly, MA, USA), phosph-p65 (Cat.: #3033, Cell Signaling), Bcl-2 (Cat.: #12789-1-AP, Protein Tech Group, Wuhan, China), Bax (Cat.: #50599-2-Ig, Protein Tech Group), IκB-α (Cat.: #ab7217, Abcam, Cambridge, MA, USA), phosph-IκB-α (Cat.: #ab133462, Abcam), p53 (Cat.: #ab1101, Abcam), p105/p50 (Cat.: #ab32360, Abcam, Cambridge), and β-actin (Cat.: #ab8227, Abcam) overnight at 4 °C. After washing with TBST, the membranes were incubated with the secondary antibodies (Cell Signaling) for one hour. The membranes were washed with TBST three times and scanned with an imaging system (Image Quant LAS 4000 mini, GE, Piscataway, NJ, USA). The densitometry of bands was analyzed using Image Pro-plus 6.0 (Media Cybernetics, Rockville, MD, USA).

CNE cells were seeded into 6-well plate at a density of 1 × 10^6^ cells/well. Exactly 48 h after treatment with TPL (0, 4 or 8 ng/mL), IR (0, 2 or 4 Gy), or TPL plus IR, the culture medium was collected, centrifuged to remove cellular debris, and stored at −70 °C until assay for VEGF. VEGF concentration was determined using a VEGF ELISA kit (R and D Systems, Minneapolis, MN, USA) according to the manufacturer’s instructions.

### 4.8. Xenograft Tumor Model

All protocols of the animal study were approved by the institutional review committee of Guangxi Medical University, School of Medicine and the National Institutes of Health (the number of ethical approval: 201610001, Date: 1 February 2016). BalB/C nude mice (4–5 weeks old, female, weighing 13–15 g) were housed in filter-capped cages, kept in a sterile facility, and maintained in a specific, pathogen-free barrier system. Mice were fed with sterile food and chlorinated sterile water. CNE cells in the logarithmic phase were digested by trypsin and made a cell suspension of 5 × 10^7^/mL. The armpit of each mouse was fixed and exposed. Then, we used a syringe to inject 0.2 mL of cell suspension which was stored on ice into in the armpit without leakage. In situ tumor growth appeared about one week after inoculation. The animals were randomly divided into four groups (*n* = 5 for each group). Each group was treated with TPL (0.075 mg/kg per day, intraperitoneally), IR (12 Gy/1f/1d), TPL plus IR, or vehicle (PBS). The size of the transplanted tumors was measured with gauged calipers every other day, and the tumor volume was calculated using the formula: volume (*V*) = 1/2 × (length × width^2^). After 16 days, the mice were euthanized by CO_2_ asphyxiation, and the tumors were removed, weighed, and photographed.

### 4.9. Protein Extraction and Western Blot Analysis

The tumors of nude mice were dissected and homogenized, then the samples of proteins were subjected to Western blot analysis using antibodies for p65, phosph-p65, VEGF, Bcl-2, Bax, IκB-α, phosph—IκB-α, p53, p105/p50, and β-actin.

### 4.10. Statistical Analysis

Data were expressed as mean ± SD. Statistical software SPSS 13.0 was performed to analyze the data using one-way analysis of variance; Values of *p* < 0.05 were considered to be statistically significant.

## 5. Conclusions

The current study indicates TPL as a promising candidate agent for the treatment of patients with NPC, a compound that is effective in combination with IR, and its synergistic apoptosis and anti-angiogenesis effects on NPC through targeting p65 phosphorylation, especially in vivo. We believe that TPL could be a much-needed candidate as a novel chemotherapeutic agent or adjuvant for the treatment of NPC, which has significant prospects in combination with existing therapies.
